# Hemodiafiltration improves performance of 24 hour *ex situ* normothermic liver machine perfusion

**DOI:** 10.1016/j.jhepr.2026.101811

**Published:** 2026-03-04

**Authors:** Jordi Vengohechea, Amelia J. Hessheimer, Javier Muñoz, Joaquim Albiol, Marina Vendrell, Josep M. Sanahuja, Javier Salinas, Carlota Largo, Paula Patricia Burgos, Soraya Rodríguez, Aida Vaquero, Mingju Liang, Fen Huo, Constantino Fondevila

**Affiliations:** 1 General & Digestive Surgery Service, Hospital Universitario La Paz, Madrid, Spain; 2 Instituto de Investigación La Paz (IdiPAZ), Madrid, Spain; 3 Centro de Investigación Biomédica en Red de Enfermedades Hepáticas y Digestivas (CIBERehd), Madrid, Spain; 4 Department of Surgery and Surgical Specializations, University of Barcelona, Barcelona, Spain; 5 Donation & Transplantation Institute Foundation, Barcelona, Spain; 6 Anesthesiology Service, Hospital Clínic Barcelona, Barcelona, Spain; 7 Animal Experimentation Laboratory, IdiPAZ, Madrid, Spain; 8 Pediatric Cardiovascular Surgery Service, Hospital Universitario La Paz, Madrid, Spain; 9 Nikkiso Spain, S.L., Madrid, Spain; 10 Guangdong Shunde Innovation Design & Research Institute, Guangdong, PR China; 11 General Hospital of Southern Theater Command of PLA, Guangdong, PR China; 12 Autonomous University of Madrid, Madrid, Spain

**Keywords:** Liver transplantation, Machine perfusion, Renal replacement therapy, Endothelium

## Abstract

**Background & Aims:**

Performing *ex situ* normothermic machine perfusion (NMP) for ≥24 h represents an opportunity to evaluate and treat livers, but is limited by the lack of support from relevant extrahepatic organs. Incorporation of systems of renal replacement therapy, including hemodiafiltration (HDF), appears useful in this regard during prolonged *ex situ* liver NMP. This study aimed to demonstrate the impact and benefits associated with incorporation of continuous HDF during 24-h *ex situ* NMP in a relevant preclinical model, including transplantation and post-transplant follow-up.

**Methods:**

Porcine livers (n = 28) underwent 24-h *ex situ* NMP with either partial perfusate exchange at 12 h and no HDF (NHDF, n = 11) or HDF initiated 2 h after NMP start (n = 17). Biochemical, histological, endothelial, and metabolomic parameters were assessed. A subset of grafts undergoing NMP + HDF (n = 8) were transplanted into recipients.

**Results:**

Incorporation of HDF during NMP maintained stable pH and electrolyte levels, effectively preventing hypernatremia, hypochloremia, and hypocalcemia developing without HDF. HDF cleared metabolic wastes (*e.g.* urea) and inflammatory cytokines (IL-1B, IL-2, IL-6, IL-8, and IL-18), resulting in reduced injury and oxidative stress markers after 24 h (Suzuki score 0.8 ± 0.4 HDF *vs.* 2.7 ± 1.3 NHDF, *p* <0.001). Vasoprotective endothelial response mechanisms, including KLF2 and eNOS gene and protein expression, were upregulated, whereas stellate cell activation and sinusoidal contraction were reduced among HDF-treated grafts. HDF reduced metabolomic alterations arising in livers during 24-h NMP, and adequate graft maintenance using NMP + HDF was demonstrated by full functional and metabolic recovery during post-transplant follow-up.

**Conclusions:**

Continuous HDF promotes a more physiological biochemical and metabolic environment, reduces inflammation and oxidative stress, and preserves homeostatic endothelial response mechanisms in livers undergoing 24-h *ex situ* NMP, facilitating successful transplantation in a complex preclinical model.

**Impact and implications:**

In this preclinical study, livers were normothermically perfused for 24 h *ex situ*, both with and without continuous HDF. Incorporation of HDF offered relevant improvements in numerous on-device measures, including the maintenance of physiological biochemical parameters; removal of injurious metabolic wastes; and improvement of injury and stress responses in parenchymal and nonparenchymal cells. A subset of livers were successfully transplanted and demonstrated full functional and metabolic recovery during follow-up. These findings indicate that advanced renal replacement therapies, such as HDF, are a key aspect of improving and prolonging *ex situ* normothermic liver perfusion, although there is ongoing need to develop more physiological metabolic support protocols for livers while on such devices.

## Highlights

• *Ex situ* liver perfusion lasting 24 h and longer is limited by the lack of relevant extrahepatic organ support.
• Continuous HDF can be incorporated to better support the liver during prolonged *ex situ* perfusion.
• Continuous HDF reduced injury, inflammation, and oxidative stress, and improved electrolyte and metabolic parameters.
• It also improved cytoprotective endothelial response mechanisms compared with no HDF.
• This preclinical study successfully perfused livers for 24 h *ex situ* and followed their functional and metabolic recovery in recipients.

## Graphical abstract

**Figure:**
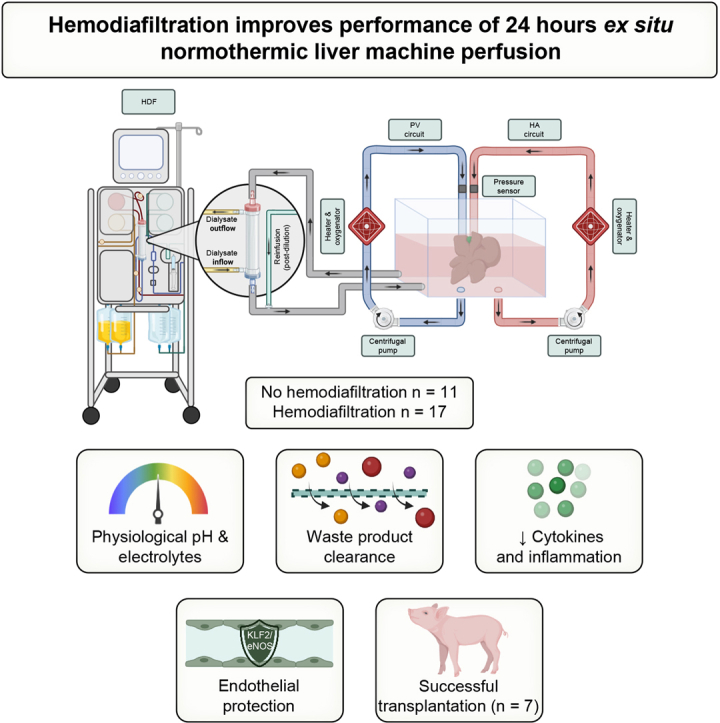

## Introduction

*Ex situ* normothermic machine perfusion (NMP) in solid organ transplantation provides an oxygenated perfusate in the 35–38 °C temperature range, in an attempt to recreate physiological conditions for the transplant allograft while in transit from the donor to the recipient’s body. Given that the liver is fully metabolically active, *ex situ* NMP offers an opportunity for graft assessment and holds important potential as a versatile platform for the application and evaluation of treatment strategies for transplant purposes and beyond.^1^ Different strategies have been preliminarily investigated in animal models and nonutilized human donor livers, including gene silencing, gene transfection, immunomodulation, and defatting in the case of steatotic livers.[2], [3], [4] Although *ex situ* NMP holds promise, there is ongoing need to establish optimal conditions to maintain organ viability for extended periods of days to weeks, depending on the therapy or intragraft processes being targeted.

At present, most liver NMP performed clinically is on devices and under protocols designed to last for only a few hours and providing far from physiological conditions.^5^ Although normothermic liver perfusion can keep a graft viable over a brief period, it creates a pseudophysiological situation, in which some liver functions might be maintained but others are notably altered.^6^^,^^7^ In this regard, a strategy to improve the conditions and duration of *ex situ* liver NMP is the addition of a system capable of clearing metabolic waste products and other injurious molecules generated during perfusion, in particular those not cleared by the liver itself. Systems of renal replacement therapy (RRT), including hemodialysis (HD), hemofiltration (HF), and hemodiafiltration (HDF), are traditionally used to provide renal support to patients with acute or chronic forms of kidney injury. When incorporated in liver NMP, they offer useful functions that can help support the hepatic microenvironment.^8^

Our research group has previously investigated liver machine perfusion, homeostasis, and regeneration in preclinical models.[9], [10], [11] Through an international collaboration, we worked to develop a prototype device for *ex situ* liver NMP, which was tested under both normothermic and hypothermic conditions in an ischemic model of porcine liver transplantation with 90 min of donor prerecovery cardiac arrest.^10^^,^^12^ Subsequently, we worked on a device and protocol for extended *ex situ* liver NMP. Here, we to describe our experience and lessons learned regarding 24-h NMP, including the impact and benefits associated with the incorporation of continuous HDF.

## Materials & methods

### Study design

With the aim of developing a device and protocol for prolonged *ex situ* liver NMP allowing for subsequent successful orthotopic liver transplantation, 24 h was selected as a perfusion duration whereby preservation would be extended beyond conventional duration and the likelihood of recipients surviving following transplantation remained high. This study was developed in a progressive and consequentially nonrandomized fashion, with increasing experience and real-time observations driving subsequent adjustments to NMP design and process. The most important among these, the decision to switch from partial perfusate exchange to incorporation of continuous HDF, defines primary study groups: (1) no HDF (‘NHDF’, n = 11): donor perfused *ex situ* for 24 h, with a partial perfusate exchange performed at 12 h; and (2) HDF (n = 17): donor livers perfused *ex situ* for 24 h, with continuous HDF initiated at 2 h (n = 9), followed by orthotopic liver transplantation in a latter subset of recipient pigs (n = 8).

### Study subjects

Male and female Landrace-Large White hybrid pigs were used as subjects, including blood donors (50 kg, n = 28), liver donors (25–30 kg, n = 28), and liver recipients (25–30 kg, n = 8). Procedures were conducted at the Center for Research on Endoscopic Surgery and Notes, Hospital Clínic Barcelona, and the Experimental Surgery Service of Hospital Universitario La Paz, in accordance with Spanish and European regulations. Both centers are accredited by the International Standards Organization (ISO9001:2015) and authorized by their respective regional authorities (CRES: ES080190036536, La Paz: ES280790001941). Experimental protocols were approved by the Catalan Department of Agriculture, Husbandry, and Fisheries; the University of Barcelona Committee on Ethics in Animal Experimentation; the Community of Madrid Directorate General of Agriculture, Livestock, and Alimentation; and the Autonomous University of Madrid Animal Welfare Body.

### Preoperative care, anesthesia, and monitoring

Animals were acclimated preoperatively and housed in pens at 18–21 °C, with 12-h:12-h light–dark cycles and free access to food and water. Food was withheld 12 h before surgery. Anesthesia was induced and maintained and study subjects monitored as described previously.^9^

### Blood donors

Via femoral vein puncture, blood was collected into CPDA-1 bags (2CD456E%, Terumo BCT Europe, N.V., Zaventem, Belgium) and stored at 4 °C, with continuous agitation until use. Before collection, 40 ml of CPDA-1 solution was withdrawn from each bag, to prevent glycemic spikes during NMP.

### Liver donors

Liver grafts were prepared *in situ*, as described previously.^9^ Liver donor blood was recovered into partially depleted CPDA-1 bags via puncture of the descending thoracic aorta until circulation ceased, at which point *in situ* liver preservation was initiated infusing cold IGL-1 (Institut Georges Lopez, Lissieu, France). Livers were recovered, weighed, and prepared. The portal vein was cannulated with a 45° angulated cannula and the hepatic artery with a flexible plastic cannula, both equipped with pressure sensor ports (Fig. S1A,B). The bile duct was cannulated with a straight silicone tube. The inferior vena cava was left open and uncannulated.

### Normothermic machine perfusion

Liver NMP was performed using a the Devocean Liver system (Guanguong Shunde Innovative Design Institute, Guangdong, China). This device includes a sterile reservoir, heat exchanger, and separate hepatic artery and portal vein perfusion circuits equipped with centrifugal pumps and long-term extracorporeal membrane oxygenators. The hepatic artery was perfused with pulsatile, high-pressure flow (100/60 mmHg) and the portal vein with continuous, low-pressure flow (4 mmHg). Oxygenation was regulated with an air–oxygen mixer (3500CP-G24, Schrist Industries, Inc., Anaheim, CA, USA), targeting 40 kPa partial pressure of oxygen and 7.35–7.45 arterial pH.

The NMP circuit was primed with porcine blood supplemented with bicarbonate, calcium, heparin, and antimicrobials. To prevent precipitate formation, calcium chloride was added before bicarbonate. Parenteral nutrition with trace elements, multivitamins, and rapid-acting insulin was administered 4 h after perfusion start. Taurocholic acid was added after 6 h. Insulin was administered as needed for hyperglycemia >6 mmol/L. Heparin was infused continuously. Table S1 provides detailed perfusate compositions and administration rates.

### Perfusate purification

In the NHDF group, 800 ml of perfusate was exchanged for 800 ml of fresh, rewarmed blood at 12 h. In the HDF group, continuous HDF was performed using the Aquarius™ System equipped with an Aquamax HF03 high-flux membrane with an effective surface area of 0.3 m^2^ and molecular weight (MW) cut-off of 40 kDa (Nikkiso Co., Ltd., Tokyo, Japan) (Table S2). HDF was run with a constant blood flow rate 100 ml/min, dialysate flow rate 250 ml/h, and replacement fluid flow delivered post-filter at 100 ml/h. Net ultrafiltration was set to 20 ml/h to account for fluid inputs and outputs, aiming for near-neutral fluid balance.^13^

### Orthotopic liver transplantation

After 24 h of NMP performed with continuous HDF, livers (n = 8) were transplanted orthotopically into recipient pigs without venovenous bypass, as per our standard protocol.^9^ Perioperative recipient management was performed as described previously.^14^ Recipient pigs were followed for up to 5 days, at which point grafts were reexamined and biopsied and animals euthanized under general anesthesia.

### Tissue sample collection and processing

Liver tissue was collected in the donor (baseline), after 24 h NMP, and 1 h and 5 days after transplant among recipients. Samples were divided into three portions: one fixed in 10% neutral-buffered formalin for paraffin embedding and histological and immunohistochemical analyses; one embedded in Tissue-Tek OCT (Sakura Finetek Europe B.V., Zoetwoude, the Netherlands) for cryosectioning; and one snap-frozen in liquid nitrogen and stored at -80 °C for RNA, protein, and metabolite extraction. Bile duct samples were collected at the same points, fixed in 10% formalin, and processed for paraffin embedding and histological evaluation. Samples were processed within 10 min of collection; integrity was confirmed by visual inspection and continuous monitoring of storage temperature for frozen sections. Samples were processed and analyzed for histological findings, immunohistochemical markers, RNA and protein expression, lipid peroxidation, and targeted metabolomics, as described in Supplemental Digital Content.

### Blood and perfusate sample collection and processing

Samples were collected from the donor at baseline and from NMP perfusate 30 min after initiation, every 2 h until 8 h, and every 4 h thereafter. Among transplant recipients, samples were collected at baseline, after reperfusion, and daily throughout follow-up. Samples were processed for standard gasometric, biochemical, and coagulation analyses, using standard automated analyzers, or centrifuged at 1,500 g for 10 min to obtain plasma aliquots, upon which cytokine analyses were performed (see Supplemental Digital Content).

### Bile sample collection and processing

Bile produced during NMP was collected under mineral oil, and samples were analyzed every 2 h for ionic composition, glucose, and pH, using an automated analyzer.

### Data processing and statistical analysis

Data are presented as mean ± SD unless otherwise specified. Continuous variables were compared using Student’s *t* test for normally distributed data. For non-normally distributed data, the Mann-Whitney *U* test was applied. For comparisons involving more than two groups or repeated measures, one-way ANOVA or Brown-Forsythe and Welch ANOVA tests were used for normally distributed data with equal or unequal variances, respectively. The Kruskal–Wallis test was used for nonparametric data. Categorical variables were compared using the chi-square test or Fisher’s exact test, as appropriate.

For metabolomic data, raw signals were normalized using median fold-change (MFC) adjustment to correct for global signal variation. Differences in tissue weights were corrected by MFC normalization. Multivariate analyses were performed using permutational multivariate analysis of variance (ADONIS) with R software package vegan. For paired samples, paired Student’s *t* test was used when normality was met, and the Wilcoxon signed-rank test was used otherwise. Correlations were assessed using Pearson’s correlation coefficient for normally distributed data and Spearman’s rank correlation for non-normal data. Metabolic pathway analysis was performed using MetaboAnalyst 6.0, which included enrichment analysis (Global Test method) and topology analysis (relative betweenness centrality method), based on the Kyoto Encyclopedia of Genes and Genomics (KEGG) library for *Sus scrofa*.

Statistical significance was set at *p* <0.05. Statistical analyses and graphical representations were performed using GraphPad Prism (V10.5.0, GraphPad Software, LLC, San Diego, CA, USA) and R software (v4.3.3, The R Foundation for Statistical Computing, Vienna, Austria).

## Results

A total of 28 liver grafts were perfused for 24 h on the NMP device, 11 without HDF (NHDF group) and 17 with HDF (HDF group). Grafts were connected to the device after 13 ± 5 min of relative *in situ* warm ischemia followed by 77 ± 16-min cold ischemia.

### Out-of-circuit HDF helps preserve portal flow dynamics and limit periportal edema developing during NMP

In the initial five cases in the HDF group, HDF was connected in parallel to the portal perfusion circuit (Fig. S3A). Derivation of flow through the former induced hemodynamic alterations requiring higher portal pressures to maintain adequate flow. Under baseline conditions, flow was maintained using a pressure-driven control algorithm; physiological portal flow rates were achieved with 2.0 ± 0.5 mmHg portal pressure. After starting HDF, derivation of some perfusate through the HDF circuit caused the portal flow to decline, obligating a switch from pressure-to flow-controlled perfusion to maintain the minimum portal flow rate. Portal circuit pressure rose to 6.1 ± 1.4 mmHg within 2 h after the start of HDF and 7.9 ± 1.3 mmHg after 16 h (*p* <0.001 for both comparisons relative to baseline). Sustained circuit pressure elevation was accompanied by graft weight gain, reaching 53.3 ± 31.2% at 24 h, primarily as a result of periportal edema formation (Fig. S3B). by contrast, the out-of-circuit HDF configuration, with HDF connected to the perfusion reservoir (Fig. S3C), maintained stable portal pressures and flows throughout the perfusion period, with no relevant fluctuations observed between early and later timepoints (Table S3). Lower portal pressures needed with this configuration led to significant reductions in graft weight gain (53.3 ± 31.2 *vs.* 15.5 ± 13.1%, *p* = 0.03) (Fig. S4).

### HDF promotes maintenance of physiological electrolyte levels and prevents progressive hypernatremia during NMP

**Fig. 1:**
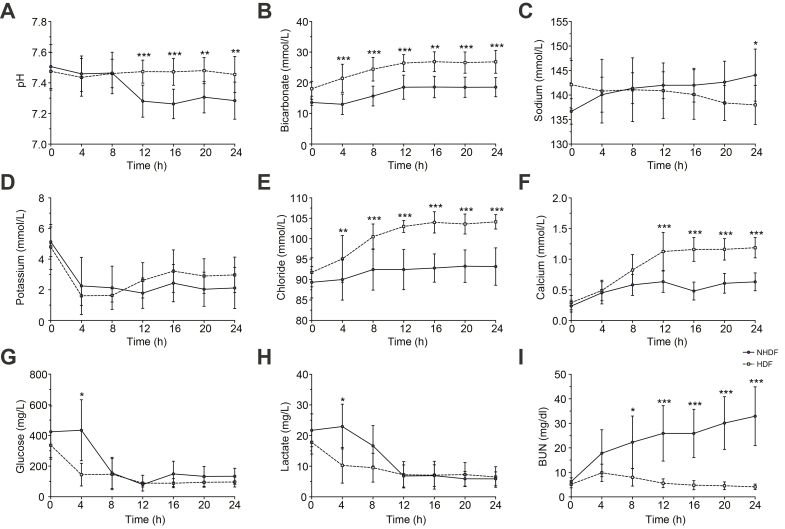
Perfusate gasometric and biochemical parameters during NMP. Application of HDF helped maintain stable, physiological levels of pH (A), bicarbonate (B), sodium (C), chloride (E), calcium (F), and BUN (I) relative to no HDF. Potassium (D), glucose (G), and lactate (H) levels were higher in both groups at the start, quickly normalized, and subsequently did not vary between groups through the remainder of perfusion. Data analyzed with one-way ANOVA for pH, bicarbonate, and chloride; Kruskal–Wallis test for glucose, lactate, sodium, potassium and BUN; and Brown-Forsythe and Welch ANOVA for calcium; ∗*p* <0.05, ∗∗*p* <0.01, ∗∗∗*p* <0.001. BUN, blood urea nitrogen; HDF, hemodiafiltration; NMP, normothermic machine perfusion.

Application of HDF helped maintain stable pH and bicarbonate levels in the perfusate, whereas the levels of both remained significantly lower throughout perfusion among grafts undergoing NMP without HDF (Fig. 1A,B). Potassium levels stabilized in both the NHDF and HDF groups (no differences). Sodium was stable throughout NMP in the HDF group but increased progressively in the NHDF group and was significantly higher at the end of NMP relative to the start (Fig. 1C,D). Furthermore HDF helped maintain physiological chloride, calcium, and osmolarity levels in the perfusate, whereas hypochloremia, hypocalcemia, and hyperosmolarity were present in the NHDF group throughout 24-h NMP (Fig. 1E,F; Fig. S5). Starting hemoglobin values were 7.9 ± 1.2 and 8.6 ± 1 g/dl (*p* = 0.63), and at the end they were 8.7 ± 1.2 and 8.6 ± 1.7 g/dl (*p* >0.99) for the NHDF and HDF groups, respectively.

### HDF supports metabolic activity and clearance of metabolic wastes produced during NMP

Stabilization of circulating glucose and lactate levels occurred more rapidly during the first 4–8 h of NMP in the HDF group, beyond which point there were no differences between NHDF and HDF grafts (Fig. 1G,H). Blood urea nitrogen (BUN) levels increased progressively throughout NMP in the NHDF group, whereas levels remained low and stable throughout NMP in HDF-treated grafts (Fig. 1I).

### HDF reduces circulating cytokine levels during NMP

**Fig. 2:**
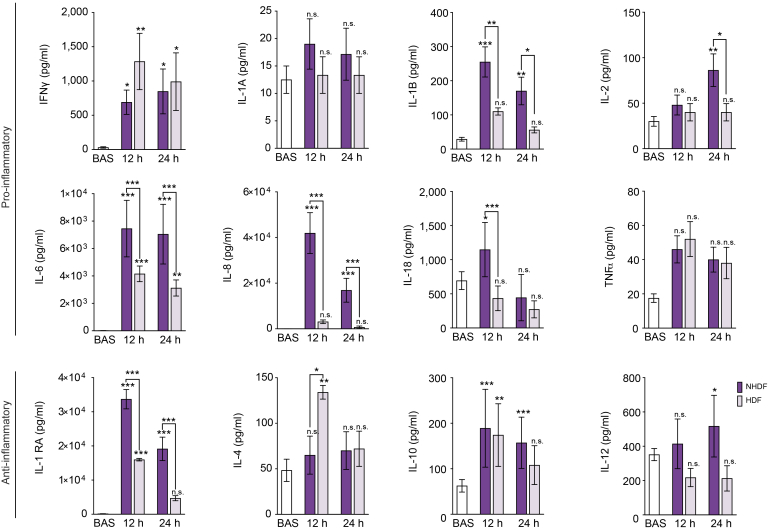
Pro- and anti-inflammatory cytokine levels during NMP. Incorporation of HDF, which nonselectively clears many cytokines, resulted in lower levels of the proinflammatory cytokines IL-18 at 12 h; IL-1B, IL-6, and IL-8 at 12 h and 24 h; and IL-2 at 24 h relative to no HDF. The anti-inflammatory cytokine IL-1RA was lower with HDF at 12 h and 24 h, whereas IL-4 was higher among grafts perfused with HDF at 12 h but not 24 h. Levels of other pro- (IFN-γ, IL-1A, and TNF-A) and anti-inflammatory cytokines (IL-10 and IL-12) rose from baseline but did not vary between groups. Data analyzed with two-way ANOVA; ∗*p* <0.05, ∗∗*p* <0.01, ∗∗∗*p* <0.001. HDF, hemodiafiltration; IFN, interferon; NMP, normothermic machine perfusion; TNF, tumor necrosis factor.

Circulating levels of many cytokines rose from baseline during the first 12 h of NMP, after which point most stabilized or declined. Incorporation of HDF, which directly and nonselectively removes many cytokines, resulted in lower levels of the following proinflammatory cytokines: IL-1B (MW 17 kDa, *p* = 0.002 and *p* = 0.01 at 12 h and 24 h, respectively), IL-2 (MW 15.5 kDa, *p* = 0.02 at 24 h), IL-6 (MW 20.9 kDa, *p* <0.001 at 12 h and 24 h), IL-8 (MW 9 kDa, *p* <0.001 at 12 h and 24 h), and IL-18 (MW 16.5 kDa, *p* <0.001 at 12 h) and the anti-inflammatory cytokine IL-1RA (MW 25 kDa, *p* <0.001 at 12 h and 24 h) relative to levels measured in the NHDF group at the same timepoints. Although the anti-inflammatory cytokine IL-4 was higher in the HDF group *vs.* NHDF at 12 h (MW 15.1 kDa, *p* = 0.01), no differences were observed at the 24-h timepoint. Levels of other pro- (interferon [IFN]-γ, MW 34.9 kDa; IL-1A, MW 17 kDa; and tumor necrosis factor [TNF]-A, MW 13.3 kDa) and anti-inflammatory cytokines (IL-10, MW 36 kDa; and IL-12, MW 70 kDa) rose marginally to significantly relative to baseline but did not vary between groups (Fig. 2).

### HDF reduces hepatocellular injury and oxidative stress during NMP

**Fig. 3:**
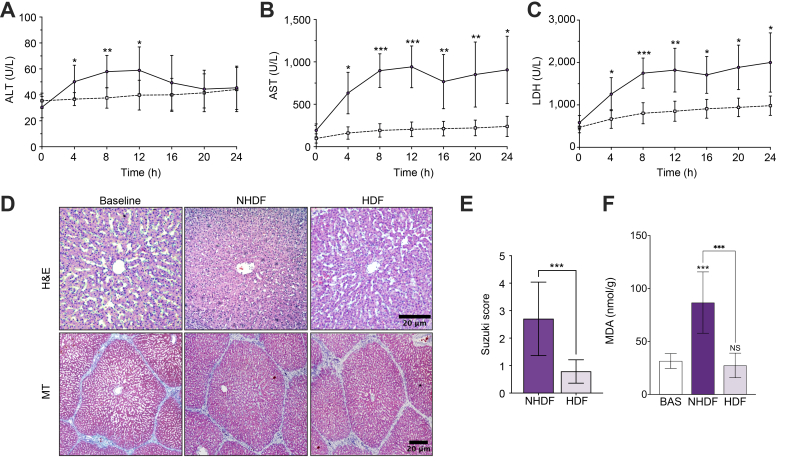
Markers of hepatocellular injury during NMP. Whereas ALT (A), AST (B), and LDH (C) dipped slightly after 12 h following partial perfusate exchange in the NHDF group, they remained higher relative to the HDF group throughout perfusion. H&E- and MT-stained tissue recovered at the end of NMP demonstrated less hepatocellular vacuolization and sinusoidal congestion (D) and lower Suzuki injury score with HDF (E). Tissue levels of MDA were also lower and almost unchanged from baseline among HDF-treated livers, whereas levels among NHDF livers were significantly higher after 24 h (F). Data analyzed with Brown-Forsythe and Welch ANOVA for AST and LDH, Kruskal–Wallis test for ALT, Mann-Whitney *U* test for Suzuki score, and one-way ANOVA for MDA; ∗*p* <0.05, ∗∗*p* <0.01, ∗∗∗*p* <0.001. ALT. alanine aminotransferase; AST, aspartate aminotransferase; HDF, hemodiafiltration; LDH, lactate dehydrogenase; MDA, malondialdehyde; MT, Masson’s trichrome; NHDF, no hemodiafiltration; NMP, normothermic machine perfusion.

During perfusion, hepatocellular injury markers, including alanine aminotransferase (ALT), aspartate aminotransferase (AST), and lactate dehydrogenase (LDH), increased progressively in the NHDF group, whereas these levels (none of which were cleared directly by HDF) remained stable with HDF. AST and LDH peaked at 4 h in HDF, stabilizing thereafter; in NHDF, they continued to rise until the end of perfusion (Fig. 3A–C). In tissue sampled after 24-h NMP, less hepatocellular injury (in particular, less hepatocellular vacuolization and sinusoidal congestion) was observed in livers in the HDF *vs.* NHDF groups (Suzuki scores 0.8 ± 0.4 *vs.* 2.7 ± 1.3, respectively, *p* <0.001) (Fig. 3D,E). MDA remained stable from the start to finish of NMP in the HDF group (31.6 ± 7 *vs.* 31.8 ± 16.8 nmol/mg, respectively, *p* >0.99), whereas levels in the NHDF group increased during NMP, both relative to baseline and to grafts undergoing HDF (90.1 ± 39 nmol/mg, *p* <0.001 for both comparisons) (Fig. 3F).

### HDF does not impact bile production but improves biliary histology during NMP

**Fig. 4:**
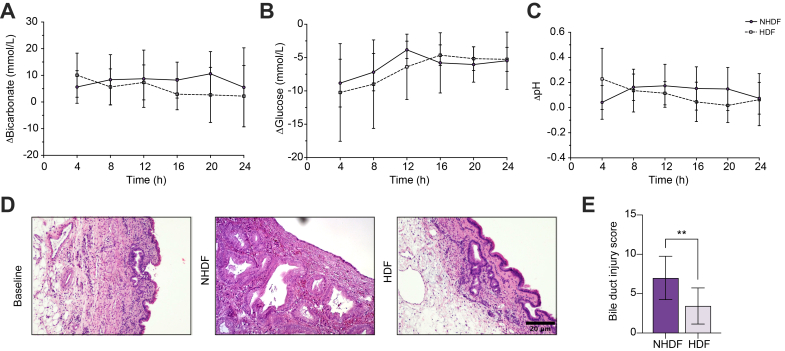
Bile composition and histology during NMP. Delta values of bicarbonate (A), glucose (B), and pH (C) demonstrated stable evolutions and no differences between HDF and NHDF. H&E-stained bile duct samples taken at baseline and end of perfusion (D) demonstrated better preservation of epithelial and mural structures in the HDF group, resulting in significantly lower histological bile duct injury score. Data analyzed with unpaired *t* test for biliary injury and one-way ANOVA test for remaining parameters; ∗∗*p* <0.01. HDF, hemodiafiltration; NHDF, no hemodiafiltration; NMP, normothermic machine perfusion.

Bile production started soon after the initiation of NMP in both groups. Taurocholic acid increased bile volume, regardless of HDF (*p* <0.001, data not shown). There were no differences between grafts undergoing NMP with and without HDF in terms of bile bicarbonate, glucose, pH, and sodium, both overall (Fig. S6) and relative to perfusate levels (Fig. 4A–C). However, histological evaluation of biliary injury indicated less microarchitectural damage among HDF-treated livers *vs.* NHDF after 24-h NMP (3 ± 2 *vs.* 7 ± 3, respectively, *p* = 0.0064) (Fig. 4D–F).

### HDF induces endothelial protection and reduces endothelial injury during NMP

**Fig. 5:**
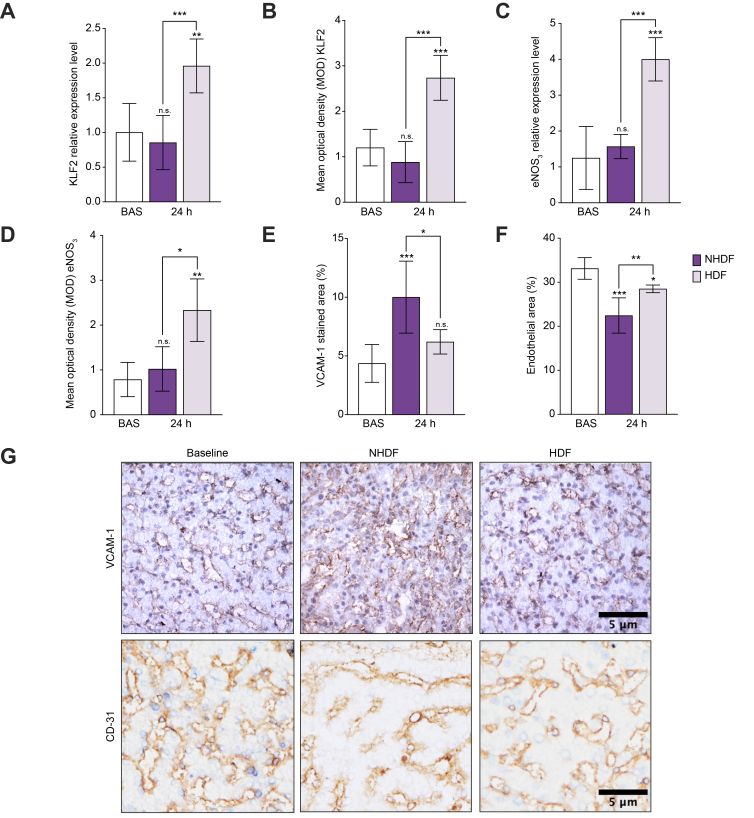
Vasoactive endothelial response and activation markers after NMP. Gene and protein expression of KLF2 (A,B) and eNOS (C,D) increased significantly by the end of 24 h in the HDF group relative to both baseline and NHDF, whereas tissue expression of VCAM-1 was higher in NHDF (E,G). Endothelial surface area was better preserved among livers treated with HDF, whereas hepatic sinusoids were collapsed among livers undergoing NMP without HDF (F,G). Full, uncropped Western blot images available in Fig. S5. Data analyzed with ordinary one-way ANOVA for KLF2 and VCAM-1, and Kruskal–Wallis test for eNOS; ∗*p* <0.05, ∗∗*p* <0.01, ∗∗∗*p* <0.001. eNOS, endothelial nitric oxide synthase; HDF, hemodiafiltration; KLF2, Krüppel-like factor 2; NHDF, no hemodiafiltration; NMP, normothermic machine perfusion; VCAM-1, vascular cell adhesion protein 1.

In the HDF group, a significant increase in both gene and protein expression of the vasodilatory mediators Krüppel-like factor 2 (KLF2) and endothelial nitric oxide synthase (eNOS) was observed at the end of perfusion compared with both baseline and the NHDF group. KLF2 gene and protein expression doubled, whereas eNOS gene and protein expression increased three-to four-fold in the HDF group (KLF2, *p* = 0.008, *p* <0.001 for remainder of comparisons). No changes were detected in the NHDF group in either KLF2 or eNOS gene or protein expression with respect to baseline (Fig. 5A–D). Tissue expression of vascular cell adhesion protein 1 (VCAM-1), an endothelial inflammatory response glycoprotein, was higher in the NHDF group at the end of NMP compared with both HDF and baseline (NHDF 24 h 10.8 ± 2.4% *vs.* HDF 24 h 6.2 ± 1.0% and baseline 4.4 ± 1.6%, *p* = 0.02 and *p* <0.001, respectively; Fig. 5E). Findings correlated with preserved endothelial surface-area integrity seen on anti-CD31 immunostaining of tissue sampled at the end of NMP performed with HDF, in contrast to notable sinusoidal collapse seen among samples taken from NHDF grafts (Fig. 5F,G).

### HDF attenuates hepatic stellate cell activation arising during NMP

**Fig. 6:**
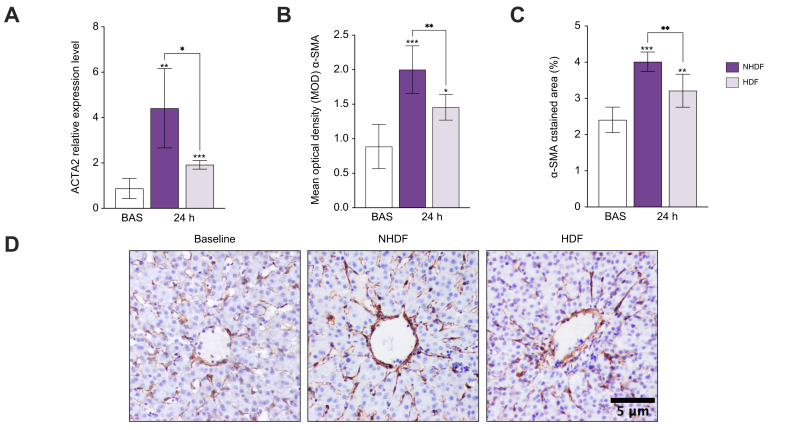
Stellate cell activation among livers undergoing NMP. Gene and protein expression of α-SMA increased relative to baseline in grafts undergoing NMP, both with and without HDF, although levels increased significantly higher in the latter (NHDF) (A,B). Immunohistochemistry confirmed greater HSC activation among NHDF livers (C,D). Western blots are available in Fig. S3 and full, uncropped Western blot images in Fig. S5. Data analyzed with Brown-Forsythe and Welch ANOVA for ACTA2 and one-way ANOVA for remaining parameters; ∗*p* <0.05, ∗∗*p* <0.01, ∗∗∗*p* <0.001. α-SMA. alpha smooth muscle actin; HDF, hemodiafiltration; HSC, hepatic stellate cell; NHDF, no hemodiafiltration; NMP, normothermic machine perfusion.

Whereas both groups showed increased *ACTA2* expression after 24-h NMP, the increase was significantly higher in the NHDF *vs.* HDF groups (4.4 ± 1.8 *vs.* 1.9 ± 0.2, respectively, *p* = 0.01) (Fig. 6A). Accordingly, alpha-smooth muscle actin (α-SMA) protein expression was also higher in the NHDF *vs.* HDF groups (2.0 ± 0.3 *vs.* 1.3 ± 0.3, respectively, *p* = 0.003) (Fig. 6B). Immunohistochemistry confirmed these findings, showing greater HSC activation among NHDF livers (Fig. 6C,D).

### HDF facilitates successful transplantation of livers undergoing NMP

**Fig. 7:**
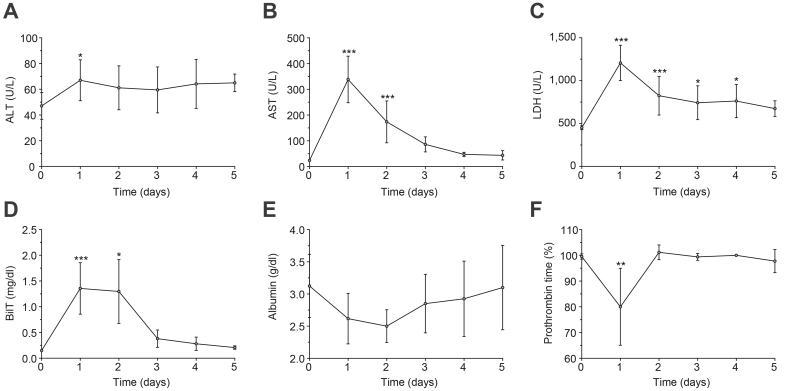
Evolution of recipients of livers transplanted after 24-h NMP+HDF. Serum ALT (A), AST (B), LDH (C), and bilirubin (D) rose marginally through the first day and normalized subsequently. Serum albumin (E) fell through the second day and normalized between days 3 and 4. Quick prothrombin time (F) fell marginally at day 1 and normalized by day 2. Data analyzed with one-way ANOVA; ∗*p* <0.05, ∗∗*p* <0.01, ∗∗∗*p* <0.001. ALT. alanine aminotransferase; AST, aspartate aminotransferase; HDF, hemodiafiltration; LDH, lactate dehydrogenase; NMP, normothermic machine perfusion.

Among eight livers that were transplanted, one recipient died during the immediate post-transplant period as a result of a hemorrhagic complication unrelated to the graft, which demonstrated initial good function. Another recipient was intentionally euthanized under anesthesia on the fourth day because of renal failure despite progressively improving liver function; at euthanasia, stenosis of the infrahepatic caval anastomosis causing renal congestion was observed. The remaining six recipients survived 5 days of follow-up, during which time livers recovered normal injury markers and synthetic function (Fig. 7).

### Metabolomic alterations arising during NMP are attenuated by HDF and reversed following transplantation

**Fig. 8:**
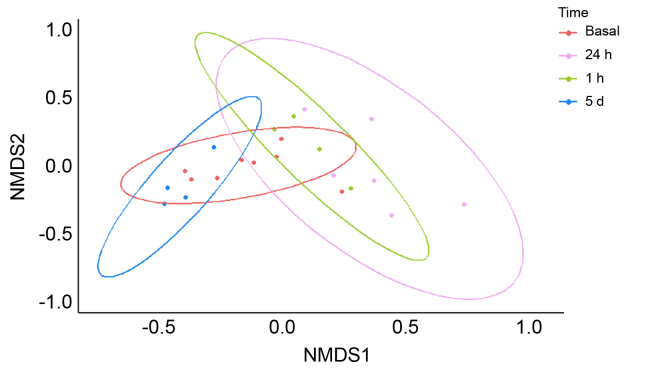
Non-metric multidimensional scaling analysis of targeted metabolomic profiles. Samples collected at baseline (red), end of NMP (pink), 1 h post-transplant (green), and 5 days post-transplant (blue) were analyzed. Points represent individual samples, and ellipses denote 95% CIs for each group. Analysis revealed a temporal shift in the hepatic metabolomic profile during perfusion, with a near-total recovery 5 days post transplant (PERMANOVA test). NMP, normothermic machine perfusion.

Targeted metabolomic analyses demonstrated that, after 24 h of perfusion, both the NHDF and HDF groups exhibited significant alterations in metabolites associated with methionine and glutathione cycles (*p* = 0.011 and *p* = 0.014, respectively). Overall metabolic profiles at the end of perfusion also differed between the two groups (*p* = 0.017), with more pronounced changes observed in the NHDF group relative to baseline. Specifically, the NHDF group had reductions in choline, methionine, *S*-adenosylhomocysteine, and methylthioadenosine, along with increased spermidine and threonine concentrations. By contrast, only *S*-adenosylmethionine was reduced in the HDF group, and other metabolites remained relatively stable throughout perfusion (Table S4). Post-transplant metabolomic analysis performed on liver biopsies taken 5 days after transplantation was consistent with restoration of physiological metabolic patterns relative to baseline (Fig. 8; Table S5).

## Discussion

This study demonstrates that incorporation of continuous HDF during 24-h NMP of porcine livers promotes a more physiological electrolyte and metabolic environment, reduces inflammation and oxidative stress, and improves injury responses, including the preservation of homeostatic endothelial response mechanisms, in both parenchymal and nonparenchymal cells. Compared with partial perfusate exchange, HDF was better able to clear nitrogenous wastes and stabilize the perfusate. NMP with partial perfusate exchange was not a control strategy *per se* but the first approach evaluated using this novel device, based on previous work describing the replacement of lysed red blood cells, repletion of bile salts, and clearance metabolic wastes.^15^ In initial experiments, we observed some perfusion destabilization at the time of exchange (fall in pH and transient shift in flows), no noticeable improvement in hemoglobin levels, and progressive increases in urea and sodium despite removal and replacement of 800 ml of the perfusate with banked porcine blood. Consequently, we switched from perfusate exchange to continuous HDF and observed better stabilization of perfusion conditions, prompting the decision to proceed with transplantation in the final set of experiments as the ultimate confirmatory test of adequate organ maintenance and viability. Post-transplant follow-up further confirmed liver metabolic recovery by the end of 5 days.

Results of this study align with those of recent systematic reviews demonstrating that incorporation of RRT offers improved electrolyte and metabolic regulation and reduced graft injury, establishing it as an essential component for prolonged *ex situ* perfusions lasting days to >2 weeks.^16^^,^^17^ HDF is currently the most advanced form of RRT available. It combines the diffusion of HD with the convection of HF. HD corrects electrolyte abnormalities, such as hypochloremia and progressive hypernatremia developing over the course of prolonged NMP seen in this and other studies.^18^^,^^19^ It also efficiently removes small toxic molecules derived from metabolism (e.g., urea), although it can also trigger complement activation and cytokine release via blood–membrane interactions. By contrast, HF uses a pressure gradient to displace plasma water across a hemofilter membrane, concomitantly dragging some solutes, such as ions, waste products, and other molecules <50 kDa, with displaced water. HF allows for elimination of both inducers and mediators of inflammation, including inflammatory cytokines and other byproducts of oxidative stress, but might be less efficient relative to HD in clearing small molecules <0.5 kDa and less efficient relative to HDF in removing larger and/or protein-bound toxins.^17^

Through combined effects of diffusion and convection, HDF lowers the levels of circulating toxins, including middle molecules and harmful protein-bound products.^20^ Lower circulating toxin levels have been seen to improve nitric oxide (NO) bioavailability and overall endothelial health and function.^21^ In this study, HDF applied during NMP reduced circulating levels of several cytokines and improved gene and protein expression of crucial endothelial vasodilators. Improved endothelial-cell responses and reduced endothelial injury seen with HDF also correlated with less stellate cell activation and sinusoidal contraction, both well-known effects of sinusoidal stress.^22^ Furthermore, out-of-circuit connection of HDF to the perfusate reservoir reduced graft (*e.g.* periportal) edema, while maintaining stable perfusion pressures.^13^

Previous studies incorporating RRT during *ex situ* liver NMP included either short 3–6-h perfusion durations (*e.g.* porcine studies) or prolonged NMP performed with neither a comparator group nor subsequent transplantation of treated grafts.^17^ Cillo and colleagues recently described a previously unprecedented 17-day perfusion of an explanted tumoral human liver using HDF.^23^ The arrangement used provided tight control of electrolytes, glucose, and nitrogenous waste products and supported bile production, synthetic activity, and microstructural integrity over 15 days, although transplantation was not performed. Eshmuminov and colleagues in Zurich transplanted porcine livers after 7 days of NMP performed on an automated device integrated with continuous HD. The group demonstrated that initial ischemia-reperfusion injury can be overcome on a perfusion device during multi-day NMP.^24^ They also used the device to perfuse a human liver from a deceased donor with sepsis for 68 h followed by successful transplantation.^25^ investigators observed in both clinical and preclinical studies that hepatocytes shrank and livers lost volume during the days of NMP.^19^^,^^25^ Such changes were observed to reverse following successful transplantation in the clinical case, causing the investigators to hypothesize that livers could be ‘put at rest’ during NMP and ‘woken up’ after transplantation.

Herein, we also observed metabolomic changes among livers undergoing NMP with HDF, changes that were largely reversed in tissue biopsies taken 5 days after successful transplantation. While NMP performed with HDF or other forms of RRT could help maintain livers in a viable state, there are important physiological processes and stimuli that are missing from even the most advanced liver NMP devices and protocols.^26^ *In vivo*, a variety of finely regulated hormones and signals endogenous to the organism but exogenous to the liver act in concert to support homeostasis and metabolic function, modify immune response and inflammation, regulate sinusoidal tone, and support regeneration of cell population subsets. These include not only insulin, glucagon, and electrolytes and nutrients commonly administered during NMP, but also primary and secondary bile acids and other gut microbe-derived metabolites, parasympathetic signaling via the vagus nerve, thyroid hormones, cortisol, sex steroids, growth hormone and IGF-1, melatonin, atrial natriuretic peptide, and bone marrow-derived progenitor cells, among others.[27], [28], [29] Although NMP obviating some or all of these might be sufficient for subsequent transplantation, it might be insufficient to study physiological responses of the liver to specific therapies or to induce processes of hepatic regeneration and repair. In this regard, the future of NMP might involve not only incorporation of extracorporeal blood purification therapies, including RRT, but also that bioreactors^30^ or even multiorgan NMP platforms.^31^

This study has limitations related to its experimental nature and limited post-transplant follow-up. Based on some anatomical limitations and lability in response to vasoactive drugs, performing orthotopic liver transplantation in the porcine model is complex.^9^ Furthermore, although it would be of interest to study more remote events and outcomes, prolonging recipient pig follow-up beyond 5–7 days increases the risk of adverse events not necessarily related to graft quality and function (*e.g.* infection or rejection). In addition, long-term animal follow-up is limited by the economic and logistical constraints of caring for recipient pigs in a long-term animal care facility. Nonetheless, the similar physiology and physically similar size and gross anatomy of the porcine liver make the porcine model the most robust and widely utilized for translation liver transplant studies. Porcine studies have provided the basis for many advances in clinical liver transplantation, including the initial implementation of liver NMP.^10^^,^^32^^,^^33^

In summary, continuous HDF applied during NMP improves numerous on-device conditions for the liver, facilitating successful transplantation even after a prolonged *ex situ* period. Although targeted metabolomic analyses indicate that HDF also helps improve the metabolic milieu of the liver during NMP relative to no HDF, ongoing alterations indicate that more research is needed to better characterize metabolic, hormonal, and other stimuli reaching the liver via the portal vein to adequately reproduce these stimuli in the *ex situ* setting.

## Abbreviations

α-SMA. alpha smooth muscle actin; ACTA2. actin alpha 2 alpha smooth muscle actin gene; ALT. alanine aminotransferase; AST, aspartate aminotransferase; BUN, blood urea nitrogen; eNOS, endothelial nitric oxide synthase; HD, hemodialysis; HDF, hemodiafiltration; HF, hemofiltration; HSC, hepatic stellate cell; IFN, interferon; ISO, International Standards Organization; KEGG, Kyoto Encyclopedia of Genes and Genomics; KLF2, Krüppel-like factor 2; LDH, lactate dehydrogenase; MDA, malondialdehyde; MFC, median fold-change; MT, Masson’s trichrome; MW, molecular weight; NHDF, no hemodiafiltration; NMP, normothermic machine perfusion; NO, nitric oxide; RRT, renal replacement therapy; TNF-A, tumor necrosis factor alpha; VCAM-1, vascular cell adhesion protein 1.

## Authors’ contributions

AJH and CF conceived and designed the study. All authors conducted the experiments and collected data. JV, AJH, AV, and CF analyzed and interpreted data. JV, AJH, and CF drafted the manuscript. All authors have read and approved the final version of the manuscript and agree to be accountable for all aspects to the work, ensuring its accuracy and integrity.

## Data availability

Data associated with this study are not publicly available but may be made available upon reasonable request to the corresponding author.

## Financial support

This study was made possible by financial support from Instituto de Salud Carlos III (PI18/0094, PI23/00364) and Guanguong Shunde Innovative Design Institute. JV was supported by a predoctoral fellowship from the University of Barcelona (PREDOCS-UB 2020). AJH was additionally supported by funding from Instituto de Salud Carlos III (PI22/00847) during the duration of the study period. These funding bodies had no role in study design, data collection and analysis, interpretation of results, or writing of the manuscript.

## Conflicts of interest

AJH has received speaker honoraria from Astellas, Medtronic, Meril, and XVIVO. CF has received speaker honoraria from Medtronic, Meril, and Olympus. The remainder of the authors have no conflicts to declare.

Please refer to the accompanying ICMJE disclosure forms for further details.

## Supplementary data

Multimeda component 1

Multimeda component 2

Multimeda component 3
