# Supplementary material for: Hemodiafiltration improves performance of 24 hour ex situ normothermic liver machine perfusion
Source: JHEP Rep. 2026 Mar 4;8(7):101811. doi: 10.1016/j.jhepr.2026.101811 (PMC13315198; doi:10.1016/j.jhepr.2026.101811)
Supplement: Multimeda component 1 [file mmc1.pdf]

Hemodiafiltration improves performance of 24 hour *ex situ* normothermic liver machine perfusion

Jordi Vengohechea, Amelia J. Hessheimer, Javier Muñoz, Joaquim Albiol, Marina Vendrell, Josep M. Sanahuja, Javier Salinas, Carlota Longo, Paula Patricia Burgos, Soraya Rodríguez, Aida Vaquero, Mingju Liang, Fen Huo, Constantino Fondevila

Table of contents

Supplementary materials and methods.. 2

Table S1...7

Table S2...9

Table S3...10

Table S4...11

Table S5...14

Fig. S1...17

Fig. S2...18

Fig. S3...19

Fig. S4...21

Fig. S5...22

Fig. S6...23

Supplementary materials and methods

Histological analysis: Paraffin-embedded tissue sections (3.5-5 μm) were cut and stained with hematoxylin and eosin (H&E) to assess general morphology and with Masson's trichrome (MT) to assess connective tissue and fibrosis. Bile duct samples were similarly processed and stained. All slides were mounted using DPX mounting medium (06522; Sigma-Aldrich Corporation, Missouri, USA). Histological sections were examined using an Olympus BX41 Brightfield/Darkfield Microscope (Olympus Corporation, Shinjaku, Japan). Images were captured at 5x and 20x magnification (NA 0.20 and 0.45, respectively) using QCapture Pro 6 software, maintaining a constant temperature of 21°C during image acquisition to ensure consistency. Liver injury was semi-quantitatively assessed using the Suzuki score, which evaluates sinusoidal congestion, hepatocellular cytoplasmic vacuolization, and parenchymal necrosis on a scale from 0 to 4 (Suzuki et al., 1991). Biliary injury was graded using a semiquantitative system described by Hansen and modified by Op den Dries (Hansen et al., 2012; Op Den Dries et al., 2014; Van Rijn et al., 2018). Scoring was performed by two independent, blinded observers to minimize bias.

Immunohistochemical analysis: Paraffin-embedded or cryopreserved tissue sections were assessed the expression of specific markers. For a-smooth muscle actin (α -SMA), sections were dewaxed, rehydrated, and subjected to heat-induced antigen retrieval in citrate buffer. After blocking, sections were incubated O.N at 4°C with a mouse monoclonal anti-human α -SMA antibody (M0851, RRID: AB_231466; Dako-Agilent Technologies, California, USA; 1:200 dilution), followed by incubation with an HRP-conjugated secondary antibody and visualization with DAB+ Substrate Chromogen System (GV825; Dako-Agilent Technologies). For VCAM-1, 5-μm cryosections were fixed in cold acetone,

blocked, and incubated O.N with a monoclonal anti-VCAM-1 antibody (Thermo Fisher Scientific, Massachusetts, USA; 1:150 dilution), followed by HRP-labeled secondary antibody and DAB+ development. For CD31, 5-μm cryosections sections were stained with a mouse anti-pig CD31 antibody (MCA1746, RRID: AB_2276640; Bio-Rad Laboratories, California, USA; 1:50 dilution) and processed similarly. For each marker, nine randomly selected fields were analyzed per section. The percentage of stained area (for α-SMA and VCAM-1) or sinusoidal lumen (for CD31) was quantified using ImageJ software (NIH, Bethesda, MD, USA). Images were converted to grayscale, background was subtracted, and the area of positive staining was measured using standardized thresholding. Results were expressed as the mean percentage of stained area per field.

RNA extraction and quantitative real-time PCR: Total RNA was extracted from snap-frozen liver tissue samples using TRIzol™ reagent (155960, Invitrogen, Carlsbad, CA, USA) according to the manufacturer's instructions. RNA concentration and purity were assessed using a NanoDrop spectrophotometer. Complementary DNA (cDNA) was synthesized by reverse transcription using a High-Capacity cDNA Reverse Transcription Kit (4374966, Applied Biosystems, Foster City, CA, USA). Quantitative real-time PCR (qRT-PCR) was performed using TaqMan Universal PCR Master Mix Expression Assays (4304437, Applied Biosystems) on a 7900HT Fast Real-Time PCR System (Applied Biosystems). Target genes included α-SMA (ACTA2; Assay ID: Ss04245588_m1, Thermo Fisher Scientific, Massachusetts, USA), Krüppel-like factor 2 (KLF2; Assay ID: Ss06942161_s1, Thermo Fisher Scientific), and endothelial nitric oxide synthase (eNOS; Assay ID: Ss03383840_g1, Thermo Fisher Scientific). Hypoxanthine-

guanine phosphoribosyltransferase (HPRT; Assay ID: Ss03388274_g1, Thermo Fisher Scientific) was used as the endogenous control for normalization. Relative gene expression was calculated using the $2^{-\Delta\Delta Ct}$ method, and results were expressed as fold change relative to the control group. All reactions were run in duplicate, and no-template controls were included to detect contamination. Only samples with A260/A280 ratios between 1.8 and 2.0 were used.

Protein extraction and Western blot analysis: Total protein was extracted from snap-frozen liver tissue samples using a Total Protein Extraction Kit (2140, Merck KGaA, Darmstadt, Germany) following the manufacturer's protocol. Protein concentration was determined using a bicinchoninic acid (BCA) protein assay kit (5000001, Bio-Rad Laboratories, Inc., California, USA). Equal amounts of protein (30 µg) were separated by SDS-PAGE on 12% precast polyacrylamide gels (4561043, Bio-Rad Laboratories) and transferred to polyvinylidene difluoride (PVDF) membranes (1704156, Bio-Rad Laboratories). Membranes were blocked with 5% non-fat dry milk in Tris-buffered saline with 0.1% Tween-20 (TBST) for 1 h at room temperature and then incubated overnight at 4°C with primary antibodies against α-SMA (M0851, RRID: AB_2314667, Dako-Aligent Technologies, California, USA, 1:200 dilution), KLF2 (BS-2772R, RRID: AB_10856812, Bioss INC., Massachusetts, USA, 1:500 dilution), eNOS (ab76198, RRID: AB_1310183, Abcam, Cambridge, UK, 1:500 dilution), and β-actin (7076, RRIS: AB_330924, Cell Signaling Technology, INC., Massachusetts, USA, 1:1000 dilution). After washing with TBST, membranes were incubated with appropriate horseradish peroxidase (HRP)-conjugated secondary antibodies (Cell Signaling Technology) for 1 h at room temperature. Protein bands were visualized using enhanced chemiluminescence (ECL) reagent (12015200001,

Hoffman-La Roche, Basel, Switzerland) and detected with a ChemiDoc MP Imaging System (Bio-Rad Laboratories). Band intensities were quantified using ImageJ software. For each target protein, the integrated optical density of the specific band was measured and normalized to the corresponding β-actin band from the same sample to control for loading variability. Results were expressed as relative optical density (O.D.). Protein loading was verified by Ponceau S staining and all Western blots included positive and negative controls.

Oxidative stress assessment: Lipid peroxidation, a marker of oxidative stress, was evaluated by quantifying malondialdehyde (MDA) levels in liver tissue using a colorimetric assay kit (MAK085-1KT, MERCK KGaA, Darmstadt, Germany). Tissue samples were incubated with thiobarbituric acid (TBA) for 60 min at 95°C. After cooling in an ice bath for 10 min, samples were transferred to a microplate, and absorbance was measured at 532 nm using a spectrophotometer. MDA concentrations were calculated from a standard curve and expressed as nmol/mg tissue. All samples and standards were run in duplicate, and a standard curve was generated for each assay.

Targeted metabolomic analysis: Liver tissue samples (10-15 mg) collected at baseline and after 24 h of perfusion were subjected to targeted metabolomics analysis for the identification and quantification of metabolites involved in the glutathione and methionine cycles. Samples were analyzed using an ultra-performance liquid chromatography system (Acquity, Waters Inc., Manchester, UK) coupled to a Time-of-Flight mass spectrometer (SYNAPT G2S, Waters Inc.) at the CIC bioGUNE Metabolomics Platform (Bizkaia Technology Park, Spain). Raw signals were adjusted by median fold-change (MFC) normalization to correct for global variations in signal intensity. Differences in tissue weights were

corrected by MFC-normalization. Metabolite identification and quantification were performed using commercially available standards and MassLynx software (Waters Inc.).

Cytokine quantification: Plasma levels of pro- and anti-inflammatory cytokine, including IFN-γ, IL-1A, IL-1B, IL-1RA, IL-2, IL-4, IL-6, IL-8, IL-10, IL-12, IL-18, TNF-A, were measured using the MILLIPLEX® Porcine Cytokine and Chemokine Magnetic Bead Panel (PCYTMAG-23K, MERCK KGaA, Darmstadt, Germany), according to the manufacturer's instructions. Briefly, plasma samples were centrifuged at 10,000 × g for 10 min at 4°C to remove debris and equilibrated to room temperature prior to analysis. Samples, standards, and quality controls were incubated with magnetic beads coated with capture antibodies in a 96-well plate overnight at room temperature with continuous shaking. After washing, biotinylated detection antibodies and Streptavidin-PE were added sequentially. Fluorescence data were acquired using a Luminex® instrument, and cytokine concentrations were calculated based on a five-parameter logistic (5-PL) regression model. All samples, standards, and controls were run in duplicate. Assay reproducibility was confirmed by including quality control samples in each plate.

Table S1

Product	Final concentration	Infusion rate	Company
Sodium Bicarbonate 1M ($NaHCO_3$)	0.03% (6.3 mL)	Priming Solution	Fresenius Kabi, Bad Homburg, Germany
Calcium Chloride 10% ($CaCl_2$)	0.04% (7 mL)	Priming Solution	B. Braun, Melsungen, Germany
Human albumin 50g/L	2.5-5 g/L (100-150 mL)	Priming Solution	CSL Behring, Pennsylvania, USA
Heparin<br>Priming solution<br>During perfusion	 5000 U<br>250000 U	 Priming Solution<br>3 cc/h	Rovi, Madrid, Spain
Metronidazole<br>Priming solution<br>12 hours	 500 mg<br>500 mg	 --<br>--	B. Braun, Melsungen, Germany
Cefoxitin<br>Priming solution<br>12 hours	 1 g<br>1g	 --<br>--	Normon, Madrid, Spain
Clinimix N14G30E	--	1 cc/h	Baxter Healthcare Corp, Illinois, USA
Supliven	0.02% (40 mL)	Supplied with Clinimix	Fresenius Kabi, Bad Homburg, Germany
Cernevit	4 vials	Supplied with Clinimix	Baxter Healthcare Corp, Illinois, USA

Short-acting insulin 100UI/ml	1 U/10 g of glucose	Hyperglycemia >6 mmol/L	Lilly, Madrid, Spain
Taurocholic acid sodium salt hydrate	1.7 mg/mL	8 cc/h	Sigma Aldrich/Merck, Missouri, USA

Pharmacological supplements and administration rates to prime and maintain 24-hour *ex situ* liver NMP.

Table S2

Component	Quantity	Concentration (for 1L of final mixture)
Sodium Chloride	6.12 g/L	6.12 g
Potassium Chloride	0.149 g/L	0.149 g
Calcium Chloride 2 H_2O	0.257 g/L	0.257 g
Magnesium Chloride 6 H_2O	0.102 g/L	0.51 g
Glucose Monohydrate	1 g/L	1 g
Sodium bicarbonate	2.94 g/L	2.94 g
Dipotassium phosphate	174.2 g/L	0.348 g
Osmolarity	296 mOsm/L	--

HDF substitution fluid composition and final concentrations used.

Table S3

Variable	NHDF (N=11)	HDF IN-LINE (N=9)	HDF OUT-OF-CIRCUIT (n=8)	*P*
PVF (mL/min)	766 (397-1107)	612 (492-766)	724 (609-893)	0.23
PVP (mmHg)	6 (4-10)	7 (6-9)	4 (3-5)	**<0.001**
HAF (mL/min)	194 (87-282)	162 (93-228)	146 (86-200)	0.23
HAP (mmHg)	72 (71-72)	71 (65-78)	71 (50-75)	0.96

Portal vein flow (PVF), portal venous pressure (PVP), hepatic artery flow (HAF), and hepatic artery pressure (HAP) measured during 24-hour *ex situ* NMP performed both with and without HDF. Portal hemodynamic measures were more stable when HDF was connected to the liver graft reservoir (out-of-circuit) as opposed to in-line in the portal vein perfusion circuit. Values are expressed as median (25-75% IQR). (Kruskal–Wallis test for PVF; Ordinary one-way ANOVA test for PVP and HAP; Brown-Forsythe and Welch ANOVA test for HAF)

Table S4

Metabolite	Baseline NHDF, Baseline HDF (pmol/mg)	NHDF (pmol/mg)	HDF (pmol/mg)	*P* Baseline *vs.* NHDF	*P* Baseline *vs.* HDF	*P* NHDF *vs.* HDF
**Glutathione Cycle**						
Glutathione (GSH)	1040.61 (837.03-1487.12), 1300.45 (1071.63-1660.31)	1291.52 (1193.92-1405.08)	1171.98 (808.14-1356.3)	0.384	0.089	0.057
Glutathione disulfide (GSGG)	6.9 (1.93-10.19), 8.78 (6.18-14.19)	8.44 (3.22-12.07)	4.93 (3.80-11.35)	0.320	0.156	**0.015**
**Methionine Cycle**						
Threonine	797.83 (634.47-891.69), 719.8 (648.7-919.06)	866.16 (800.8-921.34)	821.62 (718.94-992.78)	**0.010**	0.269	0.970

Spermine	53.71 (41.6-69.07), 41.52 (33.73-48.68)	43.99 (31.315-54.49)	35.73 (29.73-47.62)	0.193	0.991	0.450
Spermidine	76.12 (54.57-101.29), 53.56 (36.8-145.30)	125.41 (105.89-190.22)	68.98 (52.52-111.58)	**0.006**	0.563	0.130
S-Adenosyl-L-Methionine (SAMe)	59.34 (38.59-79.66), 98.62 (84.17-184.1)	91.835 (72.27-96.39)	73.07 (51.73-81.06)	0.432	**0.015**	**0.021**
S-Adenosyl-L-homocysteine (SAH)	25.92 (21.76-31.73), 19.70 (16.18-20.90)	14.7 (12.44-20.40)	18.09 (15.40-18.60)	**0.001**	0.468	**0.010**
Deoxy-5' Methylthioadenosine (MTA)	0.635 (0.48-0.70), 0.48 (0.25-0.68)	0.375 (0.22-0.48)	0.23 (0.22-0.49)	**0.018**	0.173	0.930
Methionine	14.07 (10.50-16.12), 17.62 (13.8-20.16)	7.53 (6.08-9.80)	16.74 (9.96-24.50)	**0.002**	0.916	**0.015**
Choline	796.13 (373.25-923.23), 303.185 (175.14-558.88)	304.97 (233.52-372.98)	560.38 (342.83-1447.96)	**0.010**	0.083	**<0.001**

Betaine	194.99 (123.093-278.62), 181.44 (93.36-289.02)	68.81 (7.36-103.635)	68.35 (49.11-192.49)	0.064	0.139	0.130

Concentrations of key metabolites in the methionine and glutathione cycles measured at baseline and after 24 hours of *ex situ* NMP, performed both with and without HDF. Statistical comparisons were performed between baseline and post-perfusion values within each group and between groups at 24 hours. Values are expressed as median (25-75% IQR). (Paired Student's *t* test for GSH, GSSG, MTA, SAH and Spermidine; Wilcoxon signed-rank test for the rest of the parameters).

Table S5

Metabolite	Baseline (pmol/mg)	24h NMP (pmol/mg)	1h PR (pmol/mg)	5d PR (pmol/mg)	*P* Baseline *vs.* 1h PR	*P* Baseline *vs.* 5d PR	*P* 24h NMP *vs.* 1h PR	*P* 24h NMP *vs.* 5d PR	*P* 1h PR *vs.* 5d PR
**Glutathione Cycle**									
Glutathione (GSH)	1300.45 (1071.63-1660.31)	1171.98 (808.14-1356.3)	867.39 (624.28-1084.71)	2461.82 (1963.08-2665.50)	**0.049**	0.131	0.532	0.055	**0.038**
Glutathione disulfide (GSGG)	8.78 (6.18-14.19)	4.93 (3.80-11.35)	5.26 (4.32-9.59)	29.18 (23.50-31.67)	0.354	0.133	1	0.080	0.065
**Methionine Cycle**									
Threonine	719.8 (648.73-919.06)	821.62 (718.94-992.78)	966.21 (862.43-1080.19)	908.46 (788.49-924.57)	0.150	0.531	0.439	0.784	0.365

Spermine	41.52 (33.73-48.68)	35.73 (29.73-47.62)	37.25 (36.76-46.95)	34.4 (26.09-47.25)	0.905	0.870	0.503	0.703	0.556
Spermidine	53.56 (36.8-145.30)	68.98 (52.52-111.58)	55.93 (34.49-115.76)	149.31 (106.24-182.03)	0.780	0.389	0.876	0.294	0.370
S-Adenosyl-L-Methionine (SAMe)	98.62 (84.17-184.1)	73.07 (51.73-81.06)	121.57 (60.81-156.73)	134.13 (123.31-194.78)	0.548	0.211	0.273	**0.015**	0.556
S-Adenosyl-L-homocysteine (SAH)	19.70 (16.18-20.90)	18.09 (15.40-18.60)	20.4 (16.03-23.19)	8.87 (7.31-12.01)	0.493	0.057	0.273	0.089	**0.034**
Deoxy-5' Methylthioadenosine (MTA)	0.48 (0.25-0.68)	0.23 (0.22-0.49)	0.72 (0.4-0.85)	0.39 (0.36-2.51)	0.45709818	0.88724588	0.0837210	0.15594914	0.90476190

Methionine	17.62 (13.8-20.16)	16.74 (9.96-24.50)	22.26 (21.14-26.75)	19.46 (16.62-23.11)	**0.040**	0.701	0.124	0.663	0.407
Choline	303.185 (175.14-558.88)	560.38 (342.83-1447.96)	623.5 (458.61-851.82)	162.46 (123.99-203.92)	0.130	0.080	1	**0.010**	0.063
Betaine	181.44 (93.36-289.02)	68.35 (49.11-192.49)	174.47 (167.13-208.73)	192.03 (169.53-216.03)	0.937	0.988	0.144	0.087	0.936

Concentrations of key metabolites from the methionine and glutathione cycles measured in liver tissue at baseline, after 24 hours of *ex situ* NMP, and 1 hour and 5 day post-reperfusion (PR) at transplantation. Statistical comparisons were performed between time points to assess metabolic recovery after transplantation. Values are expressed as median (25-75% IQR). (Paired Student's *t* test for GSH, GSSG; Betaine, Methionine, SAH, Spermidine and Threonine; Wilcoxon signed-rank test for the rest of the parameters).

Fig. S1

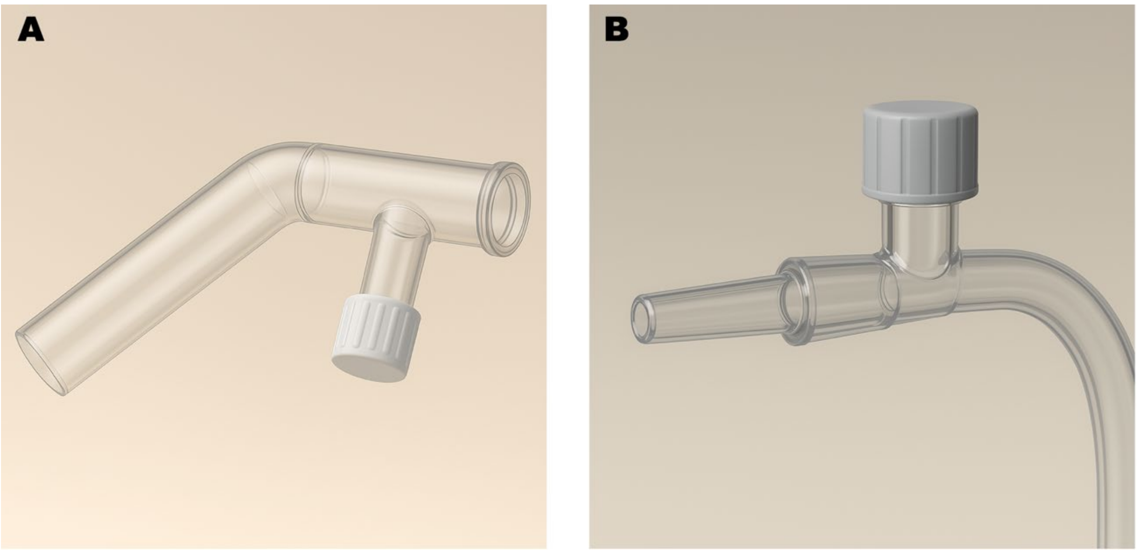

Representations of the cannulas designed for connection between the perfusion system and the portal vein (A) and hepatic artery (B) of the liver graft. The portal vein cannula features a 45° angle and pressure sensor port; the hepatic artery cannula is flexible and also equipped with a pressure sensor port.

Fig. S2

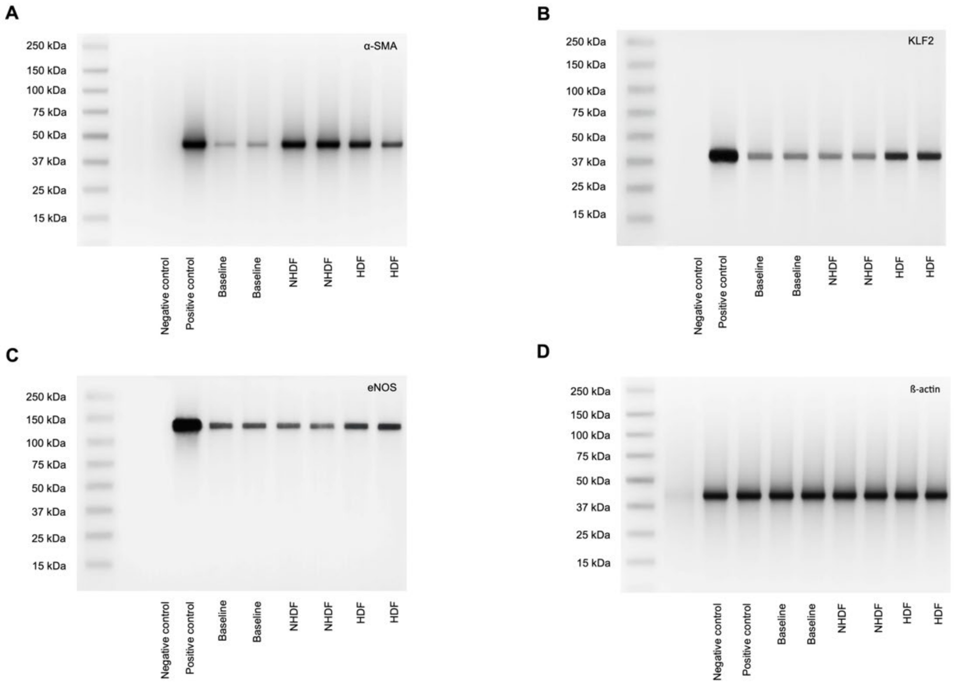

Full, uncropped Western blot images corresponding to the analyses of α-SMA (A), KLF2 (B), and eNOS (C) protein expression in liver tissue samples from NHDF and HDF groups after 24 hours of *ex situ* NMP. β-actin (D) was used as a loading control to ensure equal protein loading across samples.

Fig. S3

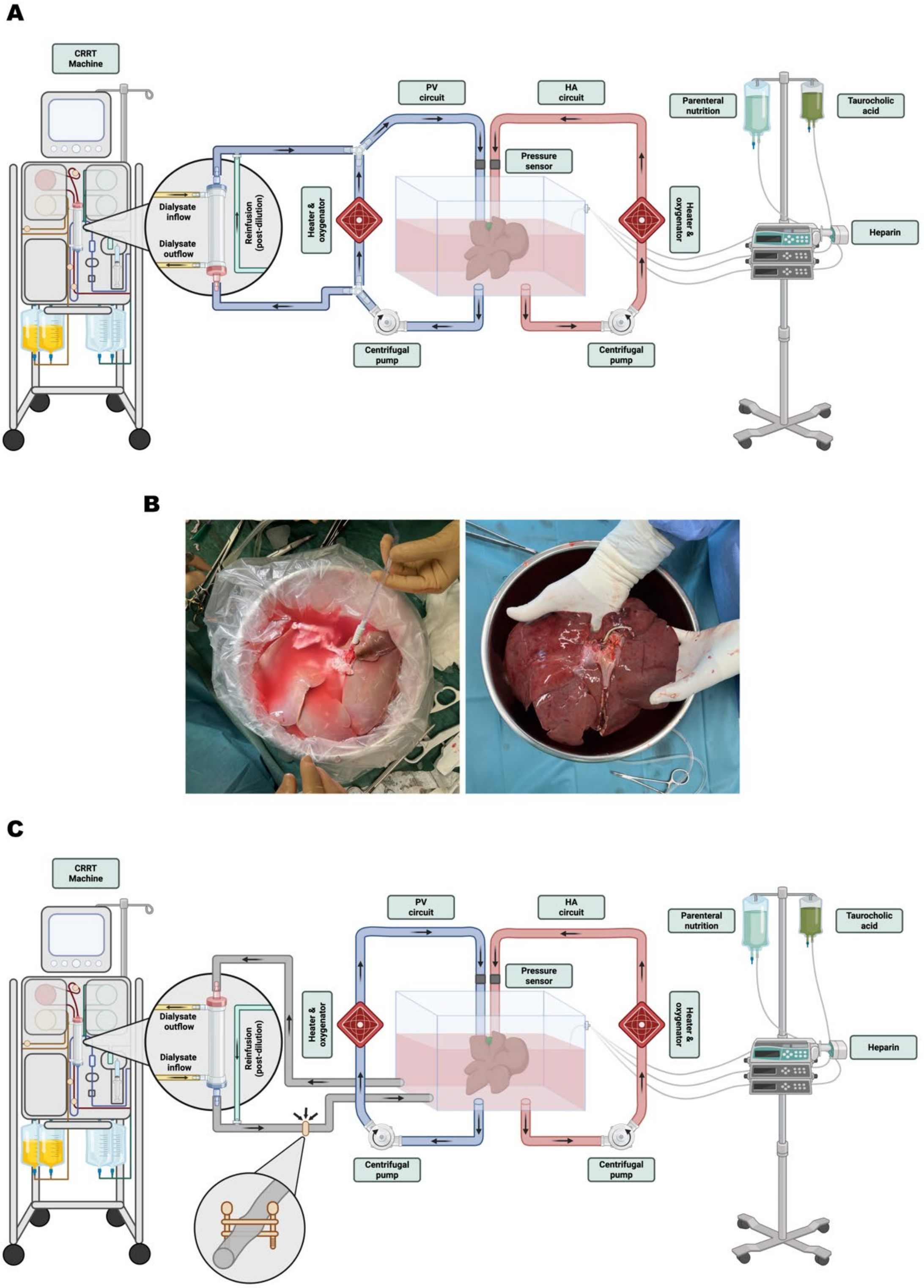

Representation of in-circuit and out-of-circuit integration of continuous renal replacement (CRRT) therapy during *ex situ* liver NMP. In the in-circuit

configuration (A), the CRRT access line is connected directly to the venous outflow of the organ, and the return line rejoins just before the cannula that connects the perfusion machine to the portal vein. This setup can increase portal venous pressure and promote periportal edema (B). In the out-of-circuit configuration (C), both the access and return lines of the CRRT are connected to the perfusion reservoir, hydraulically decoupling CRRT from the main circuit. This helps preserve hemodynamic stability and minimize pressure-induced periportal edema. The Hoffman clamp on the return line allows fine adjustment of downstream resistance. Images created using Biorender.com.

Fig. S4

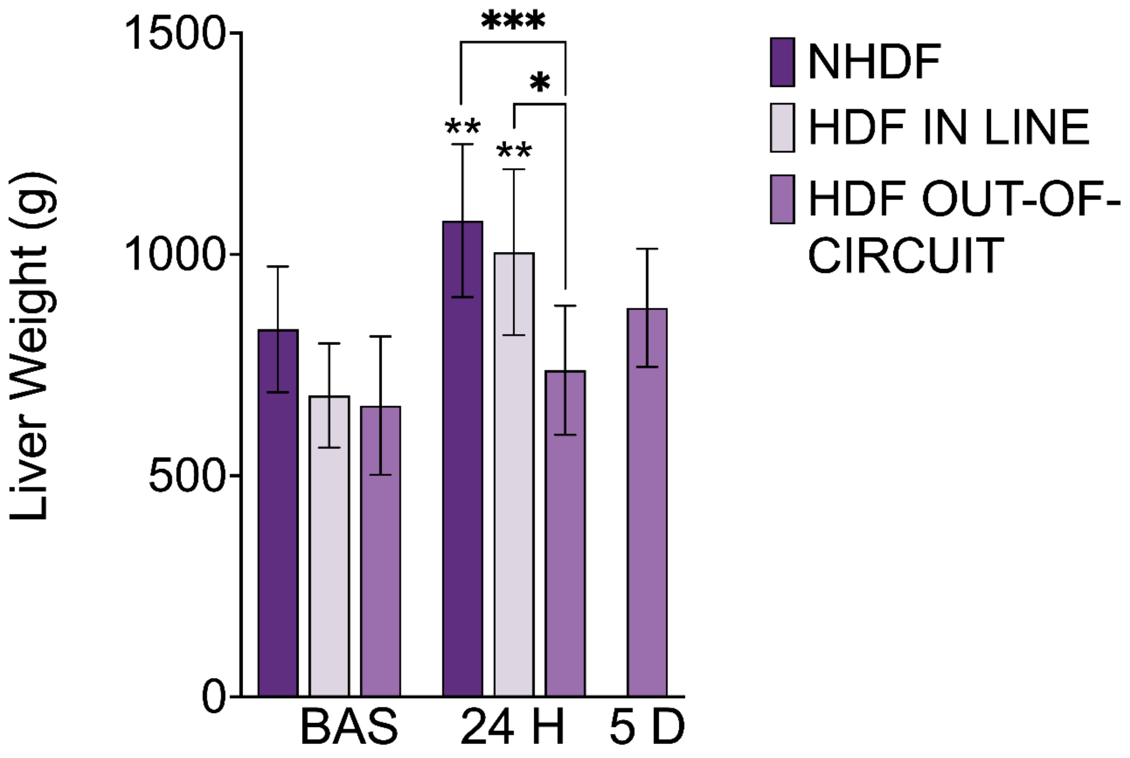

Graft weight variation following 24 hours of *ex situ* NMP perfusion and 5 days of post-transplant follow-up. In both NHDF and HDF in-line, a significant increase in graft weight was observed between baseline and the end of NMP (***P*<0.01), which was higher than the HDF out-of-circuit group (NHDF *vs.* HDF out-of-circuit ****P*<0.001, HDF in-line vs. out-of-circuit **P*=0.02). (Ordinary one-way ANOVA test**).**

Fig. S5

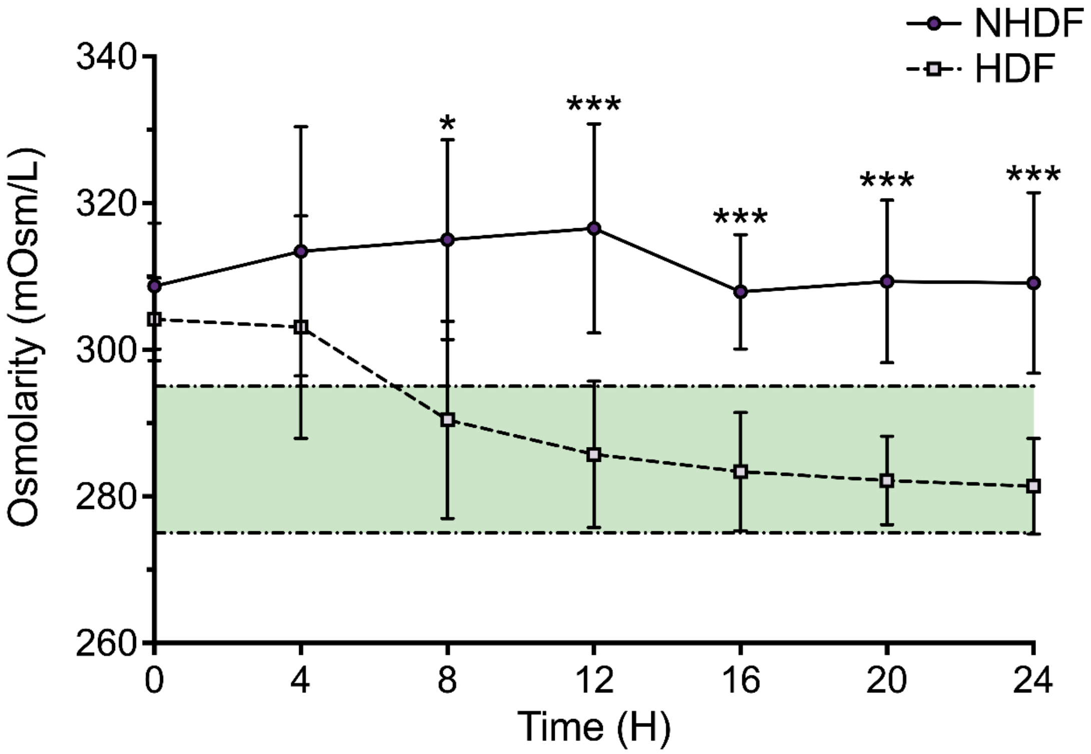

Perfusate osmolarity (mOsm/L) levels were monitored throughout 24 hours of *ex situ* NMP. The NHDF group maintained consistently elevated osmolarity values, while in the HDF group perfusate osmolarity remained largely in physiological range (275–295 mOsm/L, green shaded area). *P<0.05, ***P<0.001. (Kruskal–Wallis test).

Fig. S6

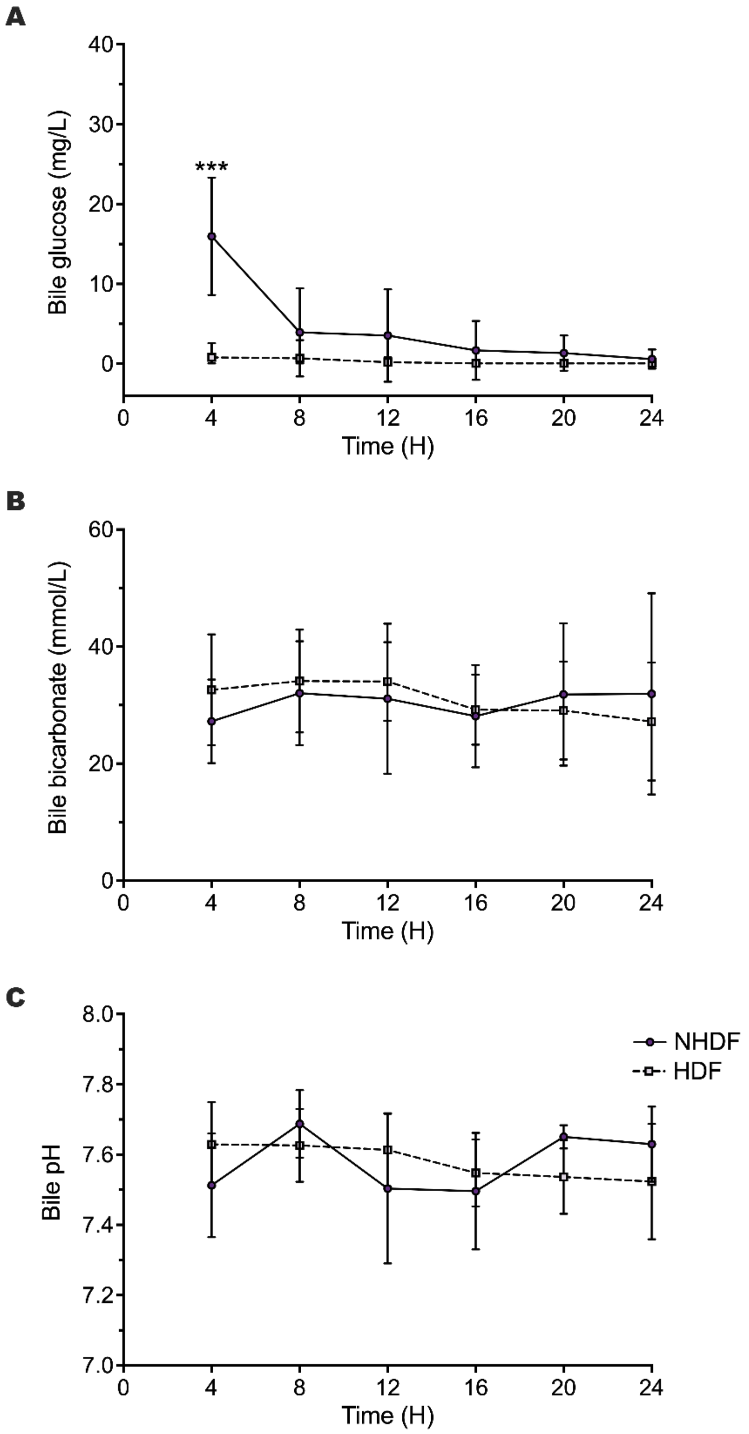
A
Bile glucose (mg/L)
***
Time (H)
B
Bile bicarbonate (mmol/L)
Time (H)
C
Bile pH
NHDF
HDF
Time (H)

Bile glucose (A), bicarbonate (B), and pH levels (C) were monitored throughout 24 hours of *ex situ* NMP. Glucose levels were higher in the NHDF group at the start of bile production (***P<0.001) but quickly decreased until there were no differences throughout the remainder of NMP. (Brown–Forsythe and Welch ANOVA test for bile glucose; Ordinary one-way ANOVA test for Bicarbonate and bile pH).
