# Supplementary material for: Hemodiafiltration improves performance of 24 hour ex situ normothermic liver machine perfusion
Source: JHEP Rep. 2026 Mar 4;8(7):101811. doi: 10.1016/j.jhepr.2026.101811 (PMC13315198; doi:10.1016/j.jhepr.2026.101811)
Supplement: Multimeda component 2 [file mmc2.docx]

**JHEP Reports**

**CTAT methods**

Tables for a “Complete, Transparent, Accurate and Timely account” (CTAT) are now mandatory for all revised submissions. The aim is to enhance the reproducibility of methods.

- Only include the parts relevant to your study
- Refer to the CTAT in the main text as ‘Supplementary CTAT Table’
- Do not add subheadings
- Add as many rows as needed to include all information
- Only include one item per row

**If the CTAT form is not relevant to your study, please outline the reasons why:**

|  |
| --- |

- 1. **Antibodies**

| **Name** | **Citation** | **Supplier** | **Cat no.** | **Clone no.** |
| --- | --- | --- | --- | --- |
| Mouse monoclonal anti-α-smooth muscle actin (α-SMA) |  | Dako-Agilent Technologies, California, USA | M0851 | 1A4 |
| Monoclonal anti-VCAM-1 |  | Thermo Fisher Scientific, Massachusetts, USA | MA5-16429 | 1.G11B1 |
| Mouse anti-pig CD31 |  | Bio‑Rad Laboratories, California, USA | MCA1746 | LCI-4 |
| Rabbit polyclonal anti-KLF2 |  | Bioss Inc., Massachusetts, USA | BS‑2772R | Not applicable (polyclonal) |
| Rabbit monoclonal anti-eNOS |  | Abcam, Cambridge, UK | ab76198 | M221 |
| Rabbit polyclonal anti‑β‑actin |  | Cell Signaling Technology, Massachusetts, USA | 7076 | Not applicable (polyclonal) |

- 1. **Cell lines**

| **Name** | **Citation** | **Supplier** | **Cat no.** | **Passage no.** | **Authentication test method** |
| --- | --- | --- | --- | --- | --- |
|  |  |  |  |  |  |

- 1. **Organisms**

| **Name** | **Citation** | **Supplier** | **Strain** | **Sex** | **Age** | **Overall n number** |
| --- | --- | --- | --- | --- | --- | --- |
| Domestic pig |  |  | Landrace–Large White hybrid | Male and Female | Age approximately 4–5 months (≈50 kg body weight) | 28 blood donors |
| Domestic pig |  |  | Landrace–Large White hybrid | Male and Female | 2–3 months (25–30 kg body weight) | 28 liver donors |
| Domestic pig |  |  | Landrace–Large White hybrid | Male and Female | 2–3 months (25–30 kg body weight) | 28 liver recipients |

- 1. **Sequence based reagents**

| **Name** | **Sequence** | **Supplier** |
| --- | --- | --- |
| TaqMan Gene Expression Assay for porcine ACTA2 (α‑SMA) | TaqMan probe/primer mix, proprietary sequence; Assay ID Ss04245588_m1 (sequence not disclosed by manufacturer) | Thermo Fisher Scientific (Applied Biosystems), Massachusetts, USA |
| TaqMan Gene Expression Assay for porcine KLF2 | TaqMan probe/primer mix, proprietary sequence; Assay ID Ss06942161_s1 (sequence not disclosed by manufacturer) | Thermo Fisher Scientific (Applied Biosystems), Massachusetts, USA |
| TaqMan Gene Expression Assay for porcine eNOS | TaqMan probe/primer mix, proprietary sequence; Assay ID Ss03383840_g1 (sequence not disclosed by manufacturer) | Thermo Fisher Scientific (Applied Biosystems), Massachusetts, USA |
| TaqMan Gene Expression Assay for porcine HPRT (endogenous control) | TaqMan probe/primer mix, proprietary sequence; Assay ID Ss03388274_g1 (sequence not disclosed by manufacturer) | Thermo Fisher Scientific (Applied Biosystems), Massachusetts, USA |

- 1. **Biological samples**

| **Description** | **Source** | **Identifier** |
| --- | --- | --- |
|  |  |  |

- 1. **Deposited data**

| **Name of repository** | **Identifier** | **Link** |
| --- | --- | --- |
|  |  |  |

- 1. **Software**

| **Software name** | **Manufacturer** | **Version** |
| --- | --- | --- |
| GraphPad Prism | GraphPad Software, LLC, San Diego, USA | 10.3.1 |
| R | The R Foundation for Statistical Computing, Vienna, Austria | 4.3.3 |
| ImageJ | National Institutes of Health (NIH), Bethesda, MD, USA | 2.16.0/1.54p |
| QCapture Pro | QImaging (Teledyne QImaging), Canada | 6.1 |
| MetaboAnalyst | Xia Lab, McGill University, Montréal, Canada | 6.0 |
| MassLynx | Waters Corporation, Manchester, UK | 4.1 |
| NanoDrop acquisition software | Thermo Fisher Scientific, Massachusetts, USA | 3.8.1 |
| 7900HT Fast Real-Time PCR System software (SDS) | Applied Biosystems, Foster City, CA, USA | 2.4 |
| ChemiDoc MP Imaging System software (Image Lab) | Bio‑Rad Laboratories, California, USA | 6.1 |

- 1. **Other (*e.g*. drugs, proteins, vectors etc.)**

| **Reagent / device** | **Description** |  |
| --- | --- | --- |
|  |  |  |

- 1. **Please provide the details of the corresponding methods author for the manuscript:**

| Name: Constantino Fondevila, MD, PhD Affiliation: General & Digestive Surgery Service, Hospital Universitario La Paz; Instituto de Investigación La Paz (IdiPAZ); CIBERehd; Autonomous University of Madrid, Madrid, Spain Address: General & Digestive Surgery Service, Hospital Universitario La Paz, Paseo de la Castellana 261, 28046 Madrid, Spain E‑mail: [constantino.fondevila@salud.madrid.org](mailto:constantino.fondevila@salud.madrid.org) |
| --- |

**2.0 Please confirm for randomised controlled trials all versions of the clinical protocol are included in the submission. These will be published online as supplementary information.**

| Not applicable. This is a preclinical porcine study and does not involve a randomised controlled clinical trial; no clinical protocol versions apply. |
| --- |
